# Improving knowledge, attitudes and practice to prevent COVID-19 transmission in healthcare workers and the public in Thailand

**DOI:** 10.1186/s12889-021-10768-y

**Published:** 2021-04-18

**Authors:** Rapeephan R. Maude, Monnaphat Jongdeepaisal, Sumawadee Skuntaniyom, Thanomvong Muntajit, Stuart D. Blacksell, Worarat Khuenpetch, Wirichada Pan-Ngum, Keetakarn Taleangkaphan, Kumtorn Malathum, Richard James Maude

**Affiliations:** 1 Faculty of Medicine Ramathibodi Hospital, Mahidol University, Bangkok, Thailand; 2 Mahidol-Oxford Tropical Medicine Research Unit, Faculty of Tropical Medicine, Mahidol University, Bangkok, Thailand; 3 Centre for Tropical Medicine and Global Health, Nuffield Department of Medicine, University of Oxford, Oxford, UK; 4 Harvard TH Chan School of Public Health, Harvard University, Boston, USA; 5 The Open University, Milton Keynes, UK

## Abstract

**Background:**

Key infection prevention and control measures to limit transmission of COVID-19 include social distancing, hand hygiene, use of facemasks and personal protective equipment. However, these have limited or no impact if not applied correctly through lack of knowledge, inappropriate attitude or incorrect practice. In order to maximise the impact of infection prevention and control measures on COVID-19 spread, we undertook a study to assess and improve knowledge, attitudes and practice among 119 healthcare workers and 100 general public in Thailand. The study setting was two inpatient hospitals providing COVID-19 testing and treatment. Detailed information on knowledge, attitudes and practice among the general public and healthcare workers regarding COVID-19 transmission and its prevention were obtained from a combination of questionnaires and observations.

**Results:**

Knowledge of the main transmission routes, commonest symptoms and recommended prevention methods was mostly very high (> 80%) in both groups. There was lower awareness of aerosols, food and drink and pets as sources of transmission; of the correct duration for handwashing; recommended distance for social/physical distancing; and about recommended types of face coverings. Information sources most used and most trusted were the workplace, work colleagues, health workers and television. The results were used to produce a set of targeted educational videos which addressed many of these gaps with subsequent improvements on retesting in a number of areas. This included improvements in handwashing practice with an increase in the number of areas correctly washed in 65.5% of the public, and 57.9% of healthcare workers. The videos were then further optimized with feedback from participants followed by another round of retesting.

**Conclusions:**

Detailed information on gaps in knowledge, attitudes and practice among the general public and healthcare workers regarding COVID-19 transmission and its prevention were obtained from a combination of questionnaires and observations. This was used to produce targeted educational videos which addressed these gaps with subsequent improvements on retesting. The resulting videos were then disseminated as a resource to aid in efforts to fight COVID-19 in Thailand and worldwide.

**Supplementary Information:**

The online version contains supplementary material available at 10.1186/s12889-021-10768-y.

## Introduction

COVID-19 is an emerging infectious disease caused by the SARS-CoV-2 virus, first discovered in the city of Wuhan, Hubei, China in December 2019 [1]. It spread to Thailand which reported the first case outside of China in January 2020 and then rapidly infected people around the world. On March 11, 2020, the World Health Organization (WHO) declared the COVID-19 outbreak a pandemic and it has since infected over 90 million people and caused 2 million deaths.

During the first wave of COVID-19 in Thailand, the government introduced a range of stringent measures to control spread including venue closures, travel restrictions, point of entry screening, quarantine, contract tracing and widespread infection prevention and control measures. These actions were very effective with Thailand suffering relatively few cases up until mid-December 2020 when a larger second wave occurred [2].

Key infection prevention and control measures for COVID-19 include social distancing, hand hygiene, use of facemasks [3] and personal protective equipment [4]. However, they have limited or no impact if not applied correctly through lack of knowledge, inappropriate attitudes or incorrect practice.

There have been a wide variety of studies to evaluate knowledge, attitudes and practice (KAP) for COVID-19 among healthcare workers and the general public [5–9]. The responses to such surveys vary widely between locations and subpopulations and a range of different scoring methods have been used. Studies have mostly focused on identifying which demographic and other variables are associated with different levels of KAP. Many have enrolled a single group of health professionals, sociodemographic/ethnic/occupational group, people with a specific chronic illness or from a single geographic location. This limits the generalizability of the findings. The few larger KAP studies covering multiple countries have included a broad range of questions. A study in 23 countries in mid-2020 found a good level of knowledge in 17.5% of participants, with this varying by country from 4.5 to 32.5% [7].

There have been few assessments of KAP focusing on the key infection prevention and control measures listed above. They have generally found levels of knowledge among healthcare workers and the public to be high and a lower proportion had good practice. For example, a study among healthcare workers in Bangladesh found 99.5% had good knowledge about PPE but only 51.7% had good practice with inadequate supply and lack of training cited by many as reasons for suboptimal practice [5]. In Nigeria, 83.7% of healthcare workers had good knowledge and 77.6% good practice towards COVID-19 prevention with good knowledge being associated with good practice [9]. Among the public, a study on COVID-19 prevention measures in Viet Nam found 92.2% to have a high knowledge level, 68.6% a positive attitude and 75.8% practiced all 6 measures to prevent virus spread with higher knowledge being associated with increased likelihood of practicing prevention measures [8]. In the public in Cameroon, 84.2% scored highly for knowledge, 69% for attitude and 60.8% for practice [10]. Evidence for educational interventions designed to improve KAP for personal protection against COVID-19 is lacking.

In order to maximise the impact of infection prevention and control measures on COVID-19 spread we undertook an intervention study to assess and improve knowledge, attitudes and practice among healthcare workers (HCW) and the general public in Thailand through educational videos.

## Methods

Members of the public (‘public’) and HCW were enrolled in July to August 2020 in Ramathibodi Hospital (a 1400 bed medical school hospital), and Somdech Phra Deparatana Medical Center (a 350 bed medical centre with a range of specilalist services), both in Bangkok, Thailand. Both study sites provide care for suspected and confirmed COVID-19. Enrolment criteria for public were any adult patient or patient attendant visiting the hospital for an outpatient visit for any reason. HCW were any hospital staff coming into contact with possible or confirmed cases of COVID-19. Both groups were deemed at potential risk of contracting COVID-19 in a healthcare setting. All participants provided written, informed consent prior to participation.

### Baseline

All participants completed a baseline questionnaire between 15th July and 30th August 2020 to assess their knowledge and attitudes about COVID-19, in particular about handwashing and use of facemasks. The questionnaire was based on the World Health Organization (WHO) COVID Behaviour Survey downloaded on 15th May 2020 and translated into Thai language. This has since been updated by the WHO and is published online [11]. Attitudes were scored on a scale of 1 (least) to 7 (most). They were also observed by experienced infection control nurses whilst using masks and washing their hands using liquid soap and water. Mask usage was assessed by observing participants putting on a surgical mask. Participants were then visited later during the same working day to observe mask removal, and determine whether and how they kept the mask for later reuse. Hand washing was assessed by covering the hands with a fluorescent dye-containing powder and then washing the hands with soap and water followed by examination under an ultraviolet light [6, 12]. To quantify hand washing effectiveness, a diagram of each hand split into 17 pre-defined areas was completed for each participant to mark any areas not covered by dye. The number of areas incompletely covered out of 34 was then counted. This method was adapted from one published previously [13].

### Video development

Using information from an interim analysis of the baseline data, a set of educational videos was produced in Thai language. This was supplemented by information from a different study conducting in-depth interviews of HCW which will be published separately. These were split into three topics to fill gaps in knowledge identified in the data: hand washing, correct use of facemasks and correct use of personal protective equipment (PPE). The videos were produced in an official audio/visual studio at Ramathibodi hospital.

### First follow-up

The same participants as at baseline viewed the videos and provided immediate feedback and suggestions on how to improve them, including technical considerations such as sound and graphics, clarifications and suggestions for additional or redundant content. They also completed the same questionnaire and observation of handwashing as at baseline to identify any changes in knowledge, attitudes or practice. The reassessment was conducted from 1 to 3 days after viewing the videos from 23rd to 30th November 2020. At that time there had been almost no local transmission of COVID for around 6 months.

### Second follow-up

A final version of the videos was produced incorporating further interim analysis and the feedback, following which they were disseminated to HCW and the general public through social media, websites and display screens. A third questionnaire was administered to the same participants 1–3 days after viewing these final videos from 1st to 5th December 2020. The third questionnaire was administered to identify any changes in KAP over time, including those from viewing the final set of changes to the videos. This was done so the impact of the final disseminated version of the videos could be studied.

### Data collection, statistical analysis and ethical approval

Data were collected on paper case record forms and entered into a secure online database. Statistical analysis was done using Microsoft Excel 2019 (Redmond, WA, USA) and GraphPad Prism version 9 (San Diego, CA, USA). Medians for items rated on a Likert scale of 1 to 7 were compared using the Wilcoxon matched pairs signed rank test. Before and after binary questionnaire results were compared using the Binomial test. The significance level was 5%. Sample size was estimated as a minimum of 100 required to be enrolled in each group (public and HCW) to have sufficient power for a binary question to detect an increase in correct responses of 8 (10%) in each group allowing for an increase of incorrect responses of 1 (1.3%) and assuming a 20% loss to follow-up.

Ethical approval was obtained from Ramathibodi Hospital Ethical Committee. All methods were performed in accordance with the relevant guidelines and regulations (Declaration of Helsinki).

## Results

One hundred public and 119 HCW were enrolled in the study (Table 1). Median age of public was 39 years with 74.0% female and median age of HCW was 37 years with 86.6% female. Occupations are shown in Table 2 with administrative and professional being the most common for public, while nurses and laboratory workers for HCW. None of either group had had confirmed COVID-19 infection, although 2.5% of HCW said they had had suspected COVID-19 infection that was not confirmed. Median household size was 3.5 for public and 3 for HCW, with 60.0% of public and 40.3% of HCW saying they lived with groups at increased risk from COVID-19. No-one reported living with or near to someone with confirmed COVID-19, although 1% of public and 6.7% of HCW reported living in the proximity of suspected COVID-19 cases.

**Table 1:** Baseline demographic and health characteristics

		Public	HCW
n		100	119
Age (years), median (IQR)		39 (32.75–47.25)	37 (30–43)
Gender	Female	74 (74.0%)	103 (86.6%)
	Male	26 (26.0%)	16 (13.4%)
Education	< 1 year	2 (2.0%)	0 (0.0%)
	1–9 years	6 (6.0%)	0 (0.0%)
	10–12 years	13 (13.0%)	7 (5.9%)
	> 12 years	79 (79.0%)	109 (91.6%)
Long-term health condition	Yes	41 (41.0%)	24 (20.2%)
Smoking	Yes	3 (3.0%)	6 (5.0%)
Alcohol	Yes	11 (11.0%)	17 (14.3%)
Have or had COVID-19	Yes, confirmed	0 (0.0%)	0 (0.0%)
	Yes, suspected not confirmed	0 (0.0%)	3 (2.5%)
	No, tested and result negative	10 (10.0%)	24 (20.2%)
	No	79 (78.9%)	75 (63.0%)
	Don’t know	8 (8.0%)	15 (12.6%)
	Blank	3 (3.0%)	2 (1.7%)
Household size, median (IQR)		3.5 (3–4)	3 (2–4)
Vulnerable groups in household	None	40 (40.0%)	71 (59.7%)
	fm,o> 60 years old	38 (38.0%)	31 (26.1%)
	Pregnant	2 (2.0%)	0 (0.0%)
	Long-term health condition	1 (1.0%)	1 (0.8%)
	Child	34 (34.0%)	29 (24.4%)
Live near case of COVID-19	Yes, confirmed	0 (0.0%)	0 (0.0%)
	Yes, suspected not confirmed	1 (1.0%)	8 (6.7%)
	No, tested and result negative	4 (4.0%)	10 (8.4%)
	No	52 (52.0%)	56 (47.1%)
	Don’t know	42 (43.0%)	45 (37.8%)
	Blank	0 (0.0%)	0 (0.0%)

**Table 2:** Occupations of participants. Administrative includes those working in an administrative role and professional includes people in an occupation requiring a qualification. HCW = healthcare worker, PBC = public

Public			HCW		
Administrative	47	(47.0%)	Nurse	43	(36.1%)
Professional	24	(24.0%)	Laboratory	39	(32.8%)
Commercial	9	(9.0%)	Nurse assistant	17	(14.3%)
Service	8	(8.0%)	Researcher/scientist	11	(9.2%)
Labourer	6	(6.0%)	Pharmacist	3	(2.5%)
Driver	4	(4.0%)	Pathology	2	(1.7%)
Unemployed	2	(2.0%)	Nutritionist	2	(1.7%)
			Phlebotomist	2	(1.7%)

### Follow-up

Eighty seven public (87%) and 100 (84%) HCW completed a questionnaire after viewing the first set of videos and 88 (88%) public and 104 (87.4%) HCW after the final set of videos. At the second follow-up visit, following feedback from participants at the first follow-up, and because of the short time interval between follow-ups, questions on attitude were not included in the questionnaire to minimize respondent fatigue. Statistical results for comparison between baseline and follow-up are presented in Table S1.

### Knowledge

**Fig. 1:**
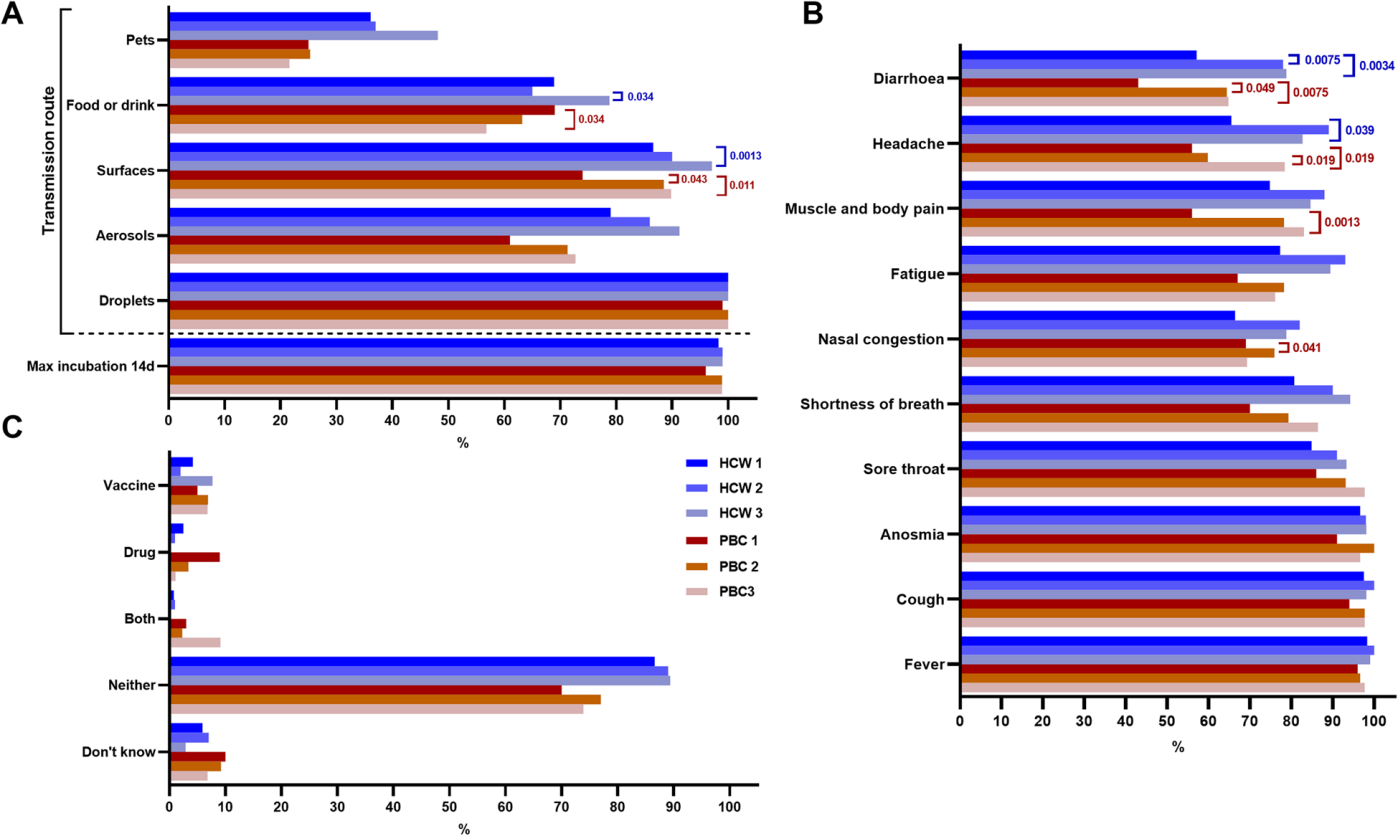
Knowledge about COVID-19 transmission sources (**a**), symptoms (**b**) and existence of treatment or vaccination for COVID-19 (**c**) before (1) and after (2) the first videos and after the final videos (3). HCW = healthcare worker, PBC = public. *P* values are shown for significant differences

Responses to the questions about general knowledge of COVID-19 are shown in Fig. 1. Overall level of knowledge was very high and similar among public and HCW with almost 100% knowing about droplet transmission and over 80% being aware of fever, cough, anosmia and sore throat as known symptoms. Among potential routes of transmission, awareness of pets was lowest followed by food and drink and aerosols. Awareness of surfaces as a possible source of COVID-19 increased after viewing the videos. Awareness of some symptoms was initially lower among the public but for diarrhoea and headache improved in both groups after viewing the videos. Awareness of muscle pain and nasal congestion improved among the public. Most people were not aware of any treatment or vaccines against COVID.

**Fig. 2:**
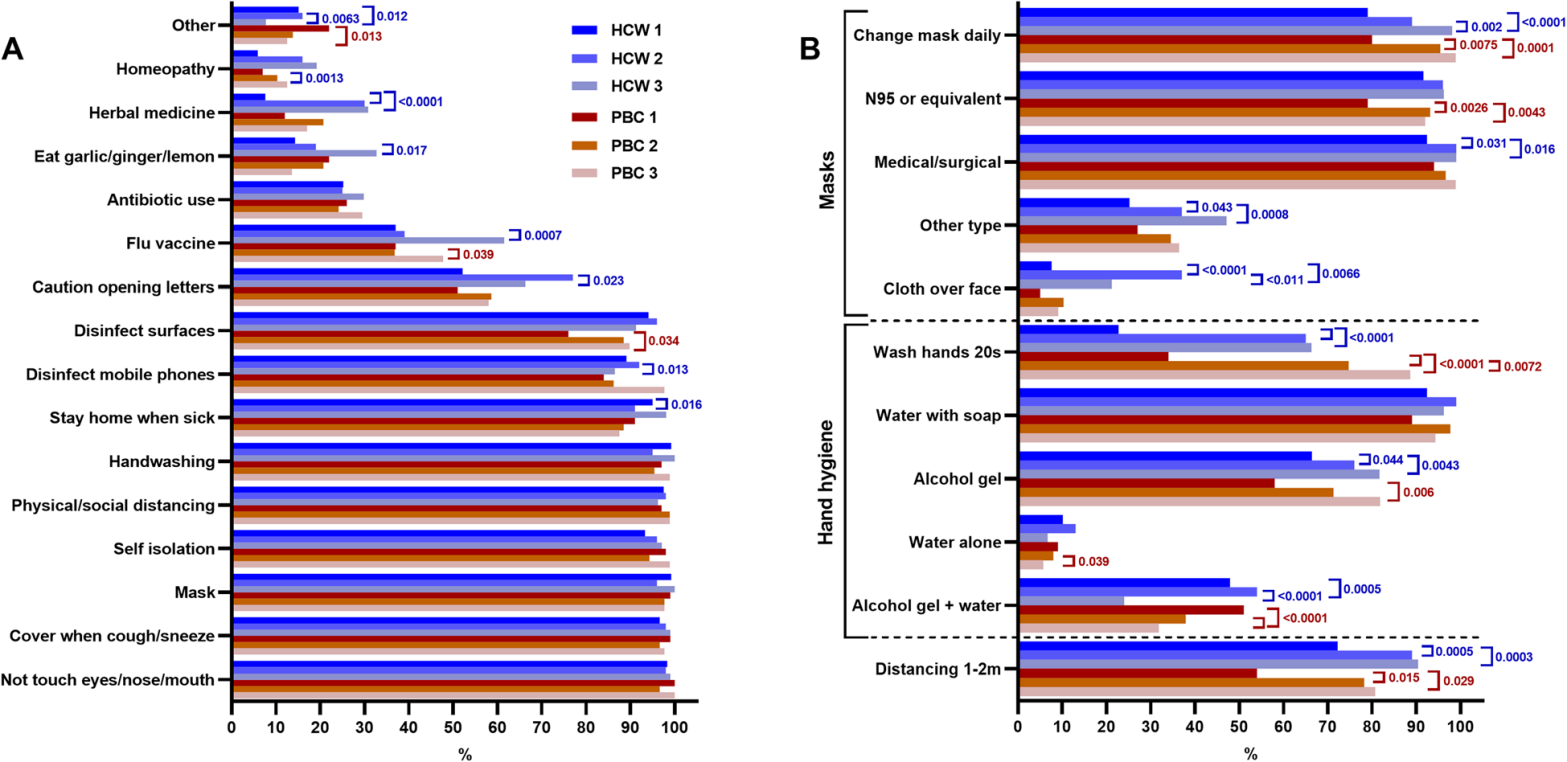
Knowledge about which COVID-19 prevention measures (**a**) and which types of mask, hand hygiene and social/physical distancing (**b**) are effective to prevent transmission of COVID-19. Results are shown for before (1) and after (2) the first videos and after the final videos (3). HCW = healthcare worker, PBC = public. *P* values are shown for significant differences

Nearly 100% of public and HCW were aware of not touching eyes/nose/mouth, covering the nose and mouth when coughing or sneezing, wearing masks, self-isolation, physical/social distancing, handwashing, disinfecting mobile phones and surfaces as measures to prevent COVID-19 transmission (Fig. 2a). Around half cited caution when opening letters and below 40% cited various other measures. Of different types of face coverings, awareness was highest of efficacy for N95 or equivalent respirators and medical/surgical masks (Fig. 2b). More than 80% knew to change their mask daily and this increased after viewing the videos in both groups. A smaller proportion knew to wash their hands for 20 s, although lower for HCW, but this also improved substantially after the videos. The majority of both groups correctly identified washing hands with soap and water or cleansing with alcohol gel as effective, the latter increasing after videos. More than 50% correctly identified 1-2 m as the correct social distancing recommendation and this also increased after the videos.

### Sources of information

**Fig. 3:**
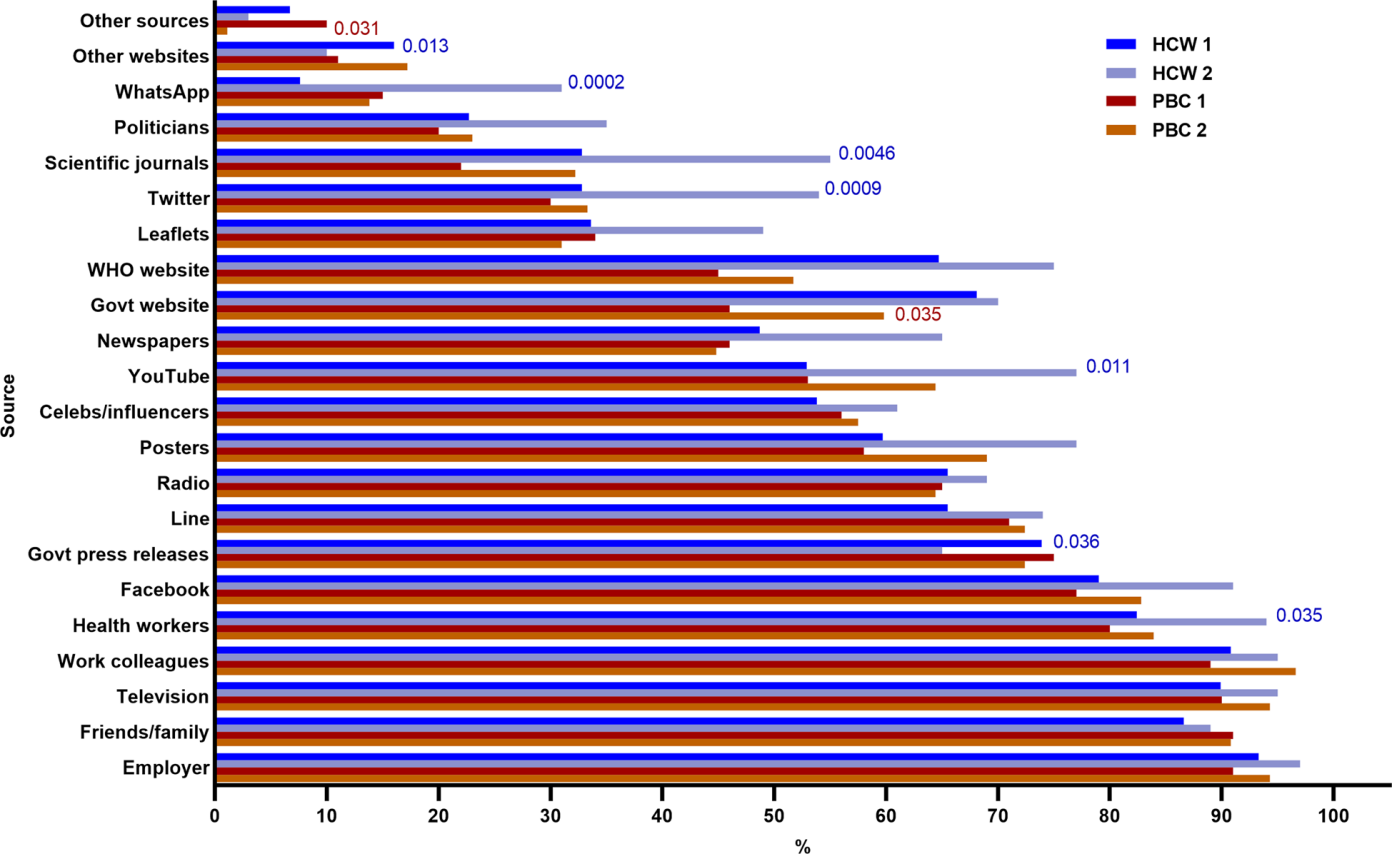
Sources of information about COVID-19 prevention stated by the public and healthcare workers (HCW) before (1) and after (2) the first set of videos. P values are shown for proportions before and after videos

Figure 3 shows where people said they obtained knowledge about preventing COVID-19. For both HCW and the public, the most commonly stated sources (> 80%) were their employer or workplace, conversations with friends and family, television, conversations with work colleagues, consultation with healthcare workers and Facebook. Twenty one percent of public and 23.5% of HCW said they had seen unclear or conflicting information. Examples included from the public concerned availability of PPE, to what degree face shields are protective and how many days people should remain in quarantine. HCW cited use of medical vs cloth masks, the amount of infection, what social distance is safe and about the need for detention of different groups of people entering the country. Facebook, Line message, television, Twitter, newspapers and conversations were among the list of sources of this unclear/conflicting information.

**Fig. 4:**
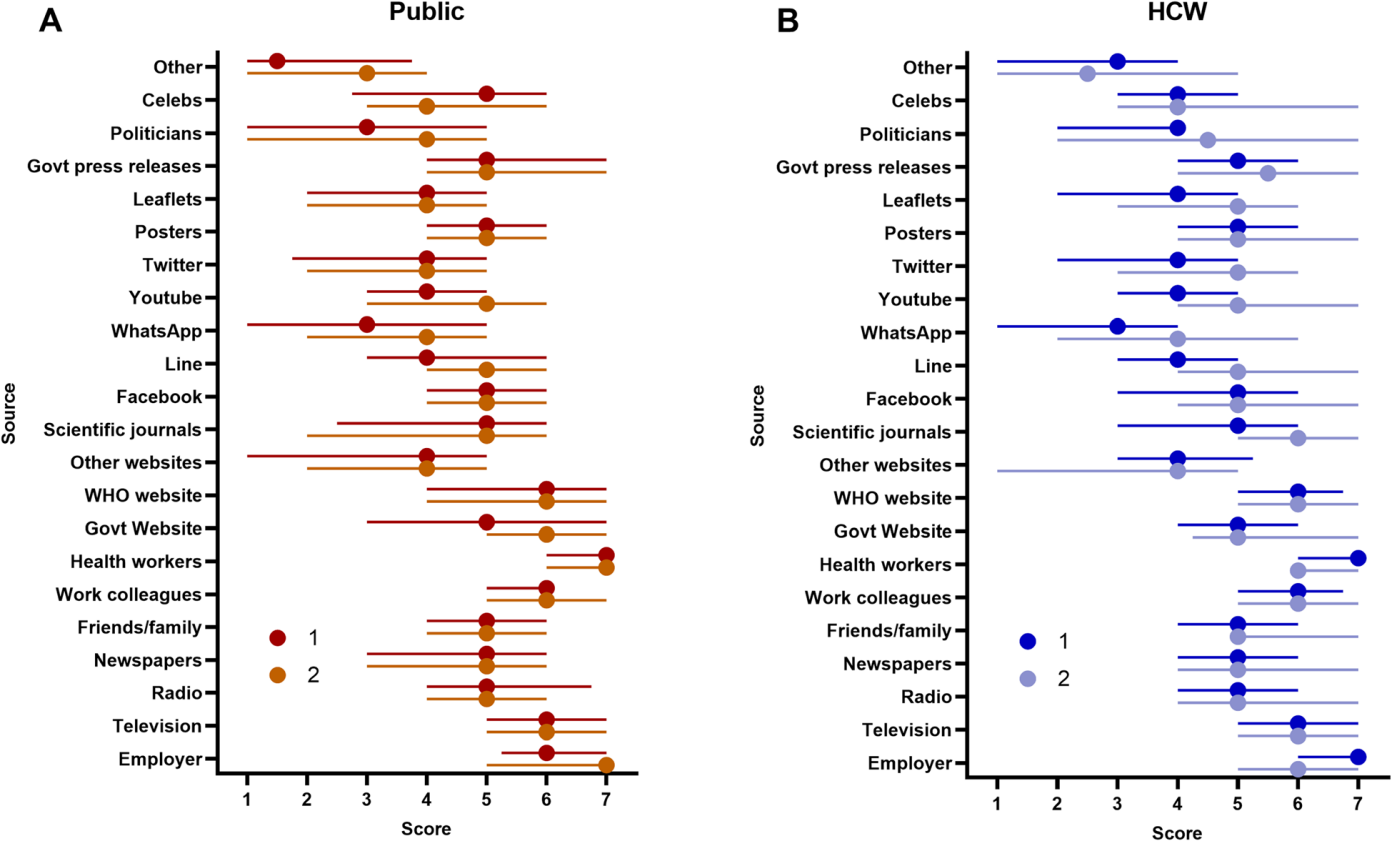
Trust in different sources of information about COVID-19 prevention assessed on a 7-point Likert scale before (1) and after (2) the first set of videos for the public and healthcare workers (HCW). Medians and interquartile ranges are shown

The most trusted sources of information among the public were healthcare workers, their employer, work colleagues and WHO and government websites and television (Fig. 4). Among HCW, the most trusted sources were healthcare workers, their employer, work colleagues, the WHO website, television and scientific journals.

The main sources of information did not change after viewing the first set of videos. The proportions of HCW and public saying they had seen unclear or conflicting information also did not change (*p* = 0.12 and 1.00, respectively). Among HCW, there were increases in the proportions using YouTube (52.9 to 77.0%), Twitter (32.8 to 54.0%), scientific journals (32.8 to 55.0%), WhatsApp (7.6 to 31.0%) and healthcare workers (82.4 to 94.0%) as sources of information and decreases in the proportions using government press releases and other websites. Among the public, the proportion using government (46.0 to 59.8%) and other websites (11.0 to 17.2%) increased. There were no major changes in the level of trust in different sources of information after viewing the first set of videos (Fig. 4).

### Attitudes

**Fig. 5:**
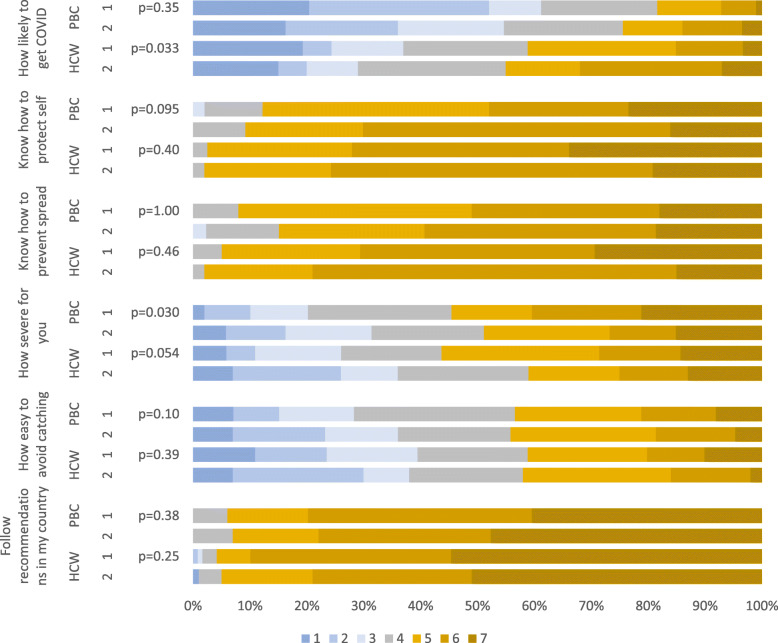
Attitudes on a Likert scale of 1 (least) to 7 (most) before (1) and after (2) viewing the first set of videos. HCW = healthcare worker, PBC = public. P values are shown for comparison of before and after video viewing

Attitudes were scored on a scale of 1 (least) to 7 (most) (Fig. 5). 95.0% of HCW and 92.0% of public scored their knowledge of preventing spread of COVID-19 as 5, 6 or 7. 96.6% of HCW and 86.0% of PBC scored themselves 5, 6 or 7 for knowing how to protect themselves from COVID-19 infection. 95.8% of HCW and 93% of public scored themselves 5, 6 or 7 for following recommendations in their country to prevent spread. Rating their ease of avoiding becoming infected with COVID-19, 41.2% of HCW and 43.0% of public scored this 5, 6 or 7 and for likelihood of becoming infected 41.2% for HCW and 18.0% of public scored 5 to 7. Severity if they were infected with COVID-19 was rated at 5, 6 or 7 by 56.3% of HCW and 54% of public.

Following viewing of the videos, there was a significant increase in scores among HCW of the likelihood of them contracting COVID-19 with those rating themselves 6 or 7 out of 7 increasing from 15.1 to 32.0% (*p* = 0.033). There was a decrease in scores for anticipated severity of COVID-19 among the public those scoring themselves 6 or 7 decreasing from 40.0 to 26.4% (*p* = 0.030). There were no other differences in attitudes between baseline and follow-up.

### Practice

Amongst the public, 27.0%, and amongst HCW, 15.1%, said they had not washed their hands when it was necessary because of not having the right materials or facilities available. For masks, 14.0% of public and 4.2% of HCW said they had not worn a mask at some time when necessary as they did not have one with them.

**Fig. 6:**
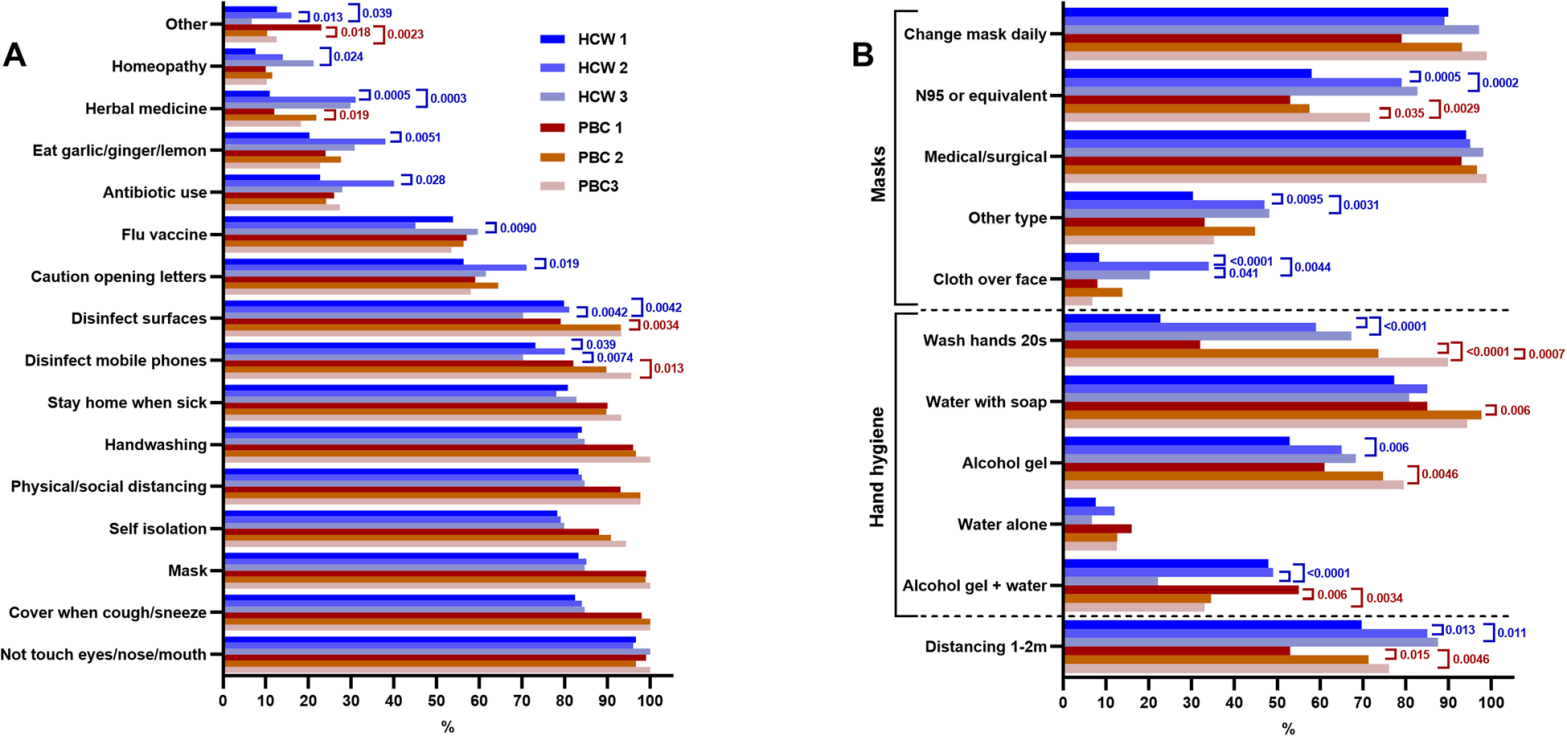
Stated practices for COVID-19 prevention measures (**a**) and which types of mask, hand hygiene and social/physical distancing (**b**) are used by participants to prevent transmission of COVID-19. Results are shown for before (1) and after (2) the first videos and after the final videos (3). HCW = healthcare worker, PBC = public. P values are shown for significant differences

Figure 6 shows the stated practices of people to prevent COVID-19 transmission, as compared to their knowledge in Fig. 3. Overall the patterns of proportions of people with particular practices were similar to those for knowledge, however for many of the most widely used measures, the proportions were lower for practice, particularly among HCW. In particular, use of N95 respirators by HCW was noticeably lower than awareness with 58% saying they used them but 91.6% saying they are effective (*p* < 0.0001) at baseline.

**Fig. 7:**
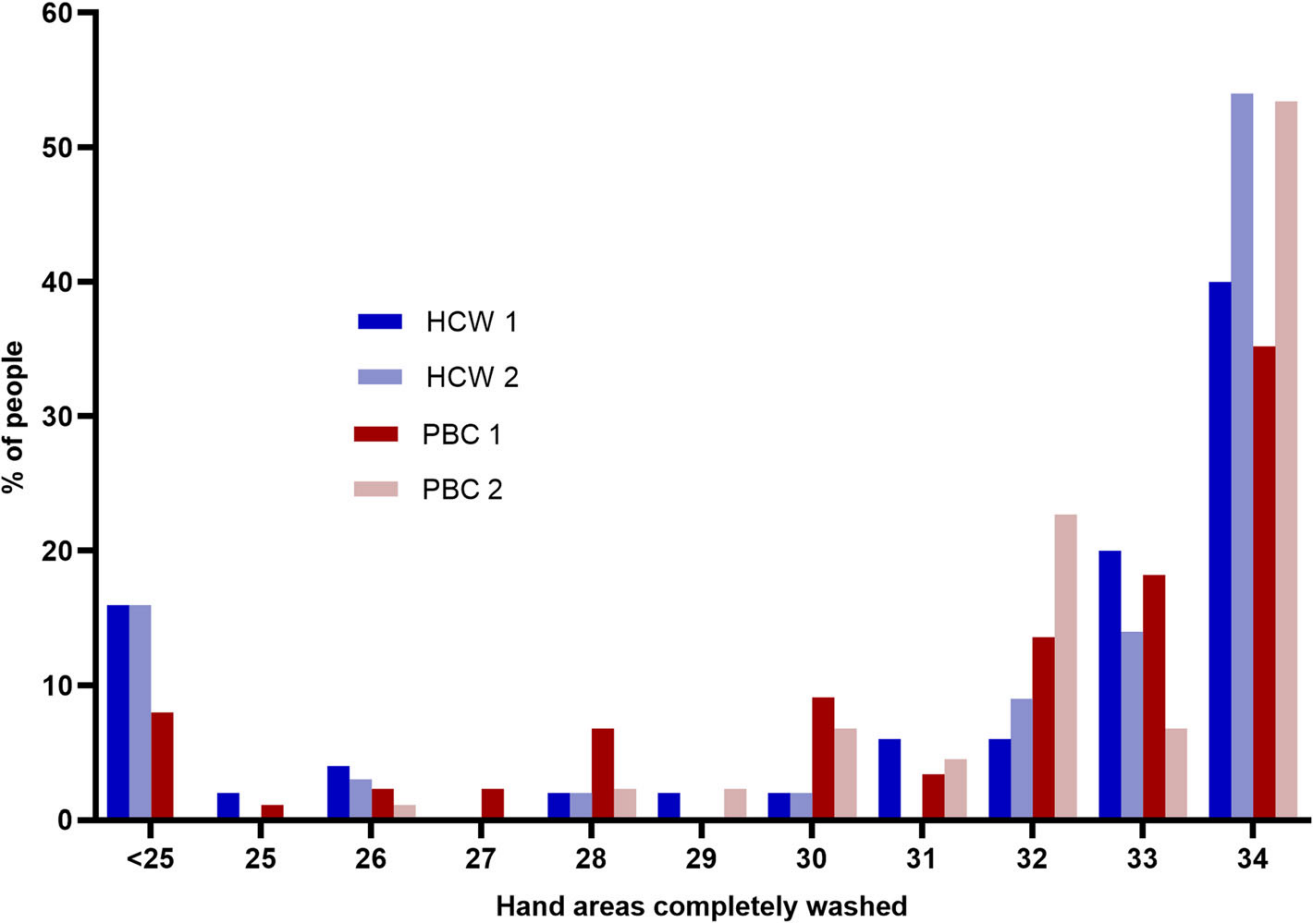
Proportion of healthcare workers (HCW) and public (PBC) who correctly washed their hands by count of areas out of 34, before (1) and after (2) watching the first set of videos

**Fig. 8:**
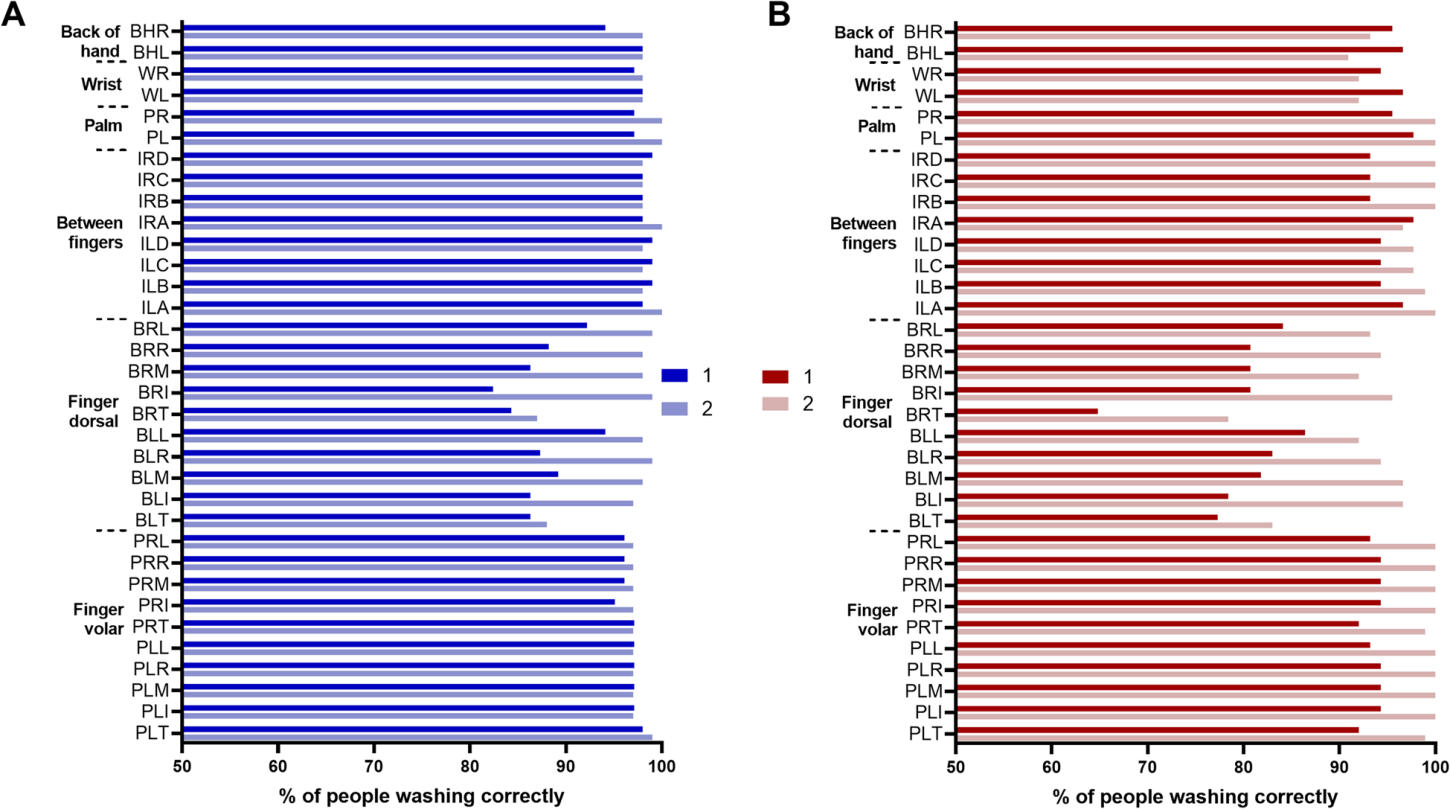
Proportion of healthcare workers (**a**) and public (**b**) who correctly washed each area of the hands before (1) and after (2) watching the first set of videos

When observed for correct wearing of a surgical mask, 100% of both public and HCW did so correctly with the mask covering both nose and mouth and the metal strip molded around the nose. When observed for correct handwashing practice, 35.2% of public and 40.0% of HCW correctly cleaned all areas of both hands (Fig. 7). The areas most poorly covered were the backs of the fingers of both hands with 45.4% of public and 55.0% of HCW washing these areas correctly (Fig. 8).

The proportions of public and HCW who did not wash their hands or wear a mask when necessary because of unavailability did not change after watching the videos.

On observation of mask wearing practice after watching the videos, 100% of both public and HCW continued to wear medical masks correctly.

On observation of handwashing practice there was an increase in the number of areas correctly washed in 65.5% of public with median (IQR) increase 4 (2–6), *p* = 0.0050, and 57.9% of HCW with median increase 2 (1–4), *p* = 0.0034 (Fig. 7). The proportion of people correctly washing each area of the hands also increased (Fig. 8) for public (*p* < 0.0001) and for HCW (*p* = 0.0002).

### Feedback on videos

Overall, participant feedback was very positive with mostly minor changes to the videos being suggested. Examples included people asking to add how to keep masks during the day, whether and how often to reuse masks, more details about handwashing steps and how to put on and take off PPE correctly to prevent self contamination.

### Dissemination

The final videos were disseminated via YouTube (https://www.youtube.com/channel/UCkwebkw5bnEVaq4Ra0qmtaw), Facebook, Line and organizational websites, as well as displayed in residential and commercial premises. Work is ongoing to produce versions with subtitles in different languages.

## Discussion

This study used questionnaires and direct observation of practice on HCW as well as the general public to assess the knowledge, attitudes and practice regarding preventing COVID-19 transmission and its prevention during the pandemic in Thailand. It identified knowledge gaps, inappropriate attitudes and suboptimal practices to target for improvement in health education programmes. In this study, they were addressed by developing and disseminating a set of educational videos focused on handwashing and mask wearing. Following participant feedback, these videos were optimized and a requested additional video on personal protective equipment for healthcare workers was added. The videos were then disseminated for viewing by the general public and healthcare professionals.

There have been many studies to evaluate knowledge, attitudes and practice (KAP) for COVID-19 [5–9]. Most of these were voluntary online surveys without a follow-up assessment or an intervention. The results of the surveys vary greatly between settings and use different methods of measurement thus making them difficult to compare and combine. Most previous KAP studies for COVID-19 have been done in either the general public or healthcare workers. This study included both groups which allowed direct comparison between them. It also recruited participants by approaching them individually, thus reducing bias that may be encountered in passive voluntary recruitment in online studies [14].

The study was able to demonstrate improvements in KAP after watching the videos. Although there was no control arm, many of the items which improved were specifically covered in the videos so it is likely that at least some of the improvement was because of watching them. This was borne out by participant’s comments. The development of the videos was deliberately done collaboratively with participants to maximise impact. Developing the video content based on identified gaps from the questionnaires, observations of practice and in-depth interviews ensured its relevance to the audience. Optimisation of the content based on user feedback helped to improve the clarity and understandability. Repeating the questionnaires and observations after viewing different iterations allowed assessment of impact by identifying specific improvements in KAP.

This approach had the advantage that all participants were able to provide anonymous feedback thus maximizing the range of viewpoints obtained. However, it only provided space for brief comments without opportunity for more detailed exploration of issues. An alternative could be to use one or more focus groups [15]. This has the potential for more in-depth discussion and candid responses among a small group of participants. However, the quality is highly dependent on the moderator, participants are generally self-selected and the process can be hijacked by outspoken individuals [16].

There have been previous intervention studies to improve KAP for other diseases similar to COVID-19. For SARS, telephone health education was able to improve knowledge of transmission routes and reduce anxiety among older adults in Hong Kong [17]. For MERS CoV in Saudi Arabi, knowledge and attitudes improved, but not practice among healthcare workers after a relatively intense educational intervention of presentations, brainstorming, interactive discussion and a short video [18]. Although effective, these methods have challenges of high resource requirements and limited scalability. This study used publicly available videos which can be rapidly and widely disseminated for maximum impact during a pandemic. The use of YouTube as the dissemination platform ensured it could be accessed through multiple types of devices at a time convenient for the audience, including mobile phones, tables, computers and smart TVs. It also made it possible for people to freely share it with others and for organisations to easily display the videos in their premises, thus widening the audience.

The method used in this study for developing the educational videos follows the principles of human centred design (HCD), namely empathy with the target communities, rapid prototyping, gathering of feedback and response iteration [19]. The process of multiple cycles of feedback from the target audience and rapid iteration in response the that feedback has been successfully applied elsewhere. This helps the audience identify with the content and ensures it meets their needs and wants [19]. After viewing the first version of the video on handwashing, some healthcare workers fed back that there should be more emphasis on washing all parts of the hands. This was from their own learnt experience having been formally trained in this. We were then able to expand this section of the video to cover this to the satisfaction of users in later feedback. HCD also includes a tolerance for failure during the design process [19]. An example from this study is that in the first video on mask wearing, the video development team did not account for the shortage of masks experienced by some participants and recommended they be disposed of after each use, as per the guidelines at the time. The participant feedback highlighted this and this led to the inclusion in the later videos of a section on how to keep a mask between uses.

The survey responses found a range of knowledge gaps, only some of which improved after watching the videos. There was low awareness of pets, food/drink and aerosols as sources of COVID-19 transmission. Although sparse, the evidence for these accumulated over the study period. Awareness was lowest for pets for which there was also the least evidence with only isolated case reports of infection in cats and dogs, as well as some wild animals: minks, tigers and lions [20–22]. The awareness of surfaces as sources of transmission increased with viewing the videos. There was a high level of awareness of the maximum incubation period of 14 days, perhaps because this had been well covered in both Thai and international media. There was also increasing awareness of the range of symptoms especially diarrhea, headache, muscle and body pain of which awareness was initially low. Awareness of fever, cough,  and anosmia was very high throughout. These three symptoms had also been highlihigh throughout. Theseghted in the popular media and government advice. Low proportions of both groups were aware of COVID-19 drug treatments or vaccinations and this did not increase during the study period. At the time of the study, there was ongoing research into both but no clear evidence of efficacy and there was little discussion of these in the national media in Thailand. Low awareness among HCW may be because they were not exposed to the ongoing research, there being no doctors included in the study. This was explored in more detail in a separate study conducting in-depth interviews among HCW.

Almost all participants correctly identified the major recommended measures to prevent COVID-19 transmission consistent with national and international guidelines. A minority identified homeopathy, herbal medicine, eating garlic/ginger/lemon and antibiotic use as effective. These measures have no evidence of clinical efficacy and are not recommended against COVID-19. For homeopathy, herbal medicine and garlic/ginger/lemon, there was an increase in the proportion believing them to be effective during the course of the study. These topics were not covered in the videos but in hindsight it may have been helpful to do so. Flu vaccine was identified as protective by an increasing proportion of both groups. Although not directly protective against COVID-19, it has been recommended to protect against coinfection with flu during the COVID-19 pandemic [23, 24]. Caution about opening letters was identified by around half of participants; although there is no evidence for this, there was some coverage of this with fake news in the national media.

Most people correctly identified facemasks should be changed daily and this increased after watching the videos, which specifically mentioned this. Most people thought N95 respirators and medical masks were protective but less than half thought other types of masks are effective. Few people thought a cloth over the face was protective. The evidence for the relative efficacy of different types of masks against COVID-19 is weak but N95 respirators and medical masks are known to prevent transmission of other respiratory viruses and bacteria [25, 26], N95 respirators are recommended for aerosolizing procedures and medical masks for general use. The recommendations for cloth or other types of masks are generally for where medical masks are not available [27–29].

Only a minority of both groups were aware of the recommended handwashing duration of 20 s [30]. There was a large increase in these proportions after watching the videos where this topic was specifically addressed. There was also an increase in the proportions of both groups who correctly identified the recommended distance for social/physical distancing, which was also in the videos [31].

The majority of people listed their employer, work colleagues and television as sources of information about COVID, more than official sources such as the government or WHO. Healthcare workers and employers were the most trusted sources. A range of social media was cited as sources but trust in these varied between different platforms with Facebook and Line being the most used and most trusted. Because of this, we chose to use both these platforms to promote our YouTube videos at the end of the project. Use of scientific journals, Twitter, YouTube and WhatsApp by healthcare workers and government websites by the general public decreased over time, perhaps because of information saturation or fatigue. The level of trust in sources generally did not change after watching the videos, although this was not included as a video topic.

Both groups rated themselves highly for level of knowledge about COVID-19 protection and spread with no change after watching the videos. However, there were many examples of improvement in knowledge from the questionnaires and observations so it is curious that people were not aware of this improvement.

There was a clear improvement of handwashing technique after watching the videos in both groups. This was covered in detail in the handwashing video and feedback was good on this particular component. Previous studies have shown video to be an effective medium for improving handwashing [32].

This study had a number of limitations. The sample size was relatively small, thus potentially limiting the representativeness. The study had to be completed quickly as the videos were needed for education and training. As data collection was done by busy healthcare staff alongside their day jobs, and during a pandemic with requirements for protective measures, the design deliberately prioritised collecting detailed responses from a fewer people over less information from larger numbers. This both minimized the number of patient-staff encounters and provided detailed and specific information on items of KAP that could be improved. Staff were encouraged to choose participants at random with broad entry criteria and there was a good range of occupations and age groups, although an excess of females. Between 12 and 16% did not return to complete a follow-up questionnaire after viewing the videos. This is despite the best efforts of the study staff with people taking leave from work, moving jobs or house and/or being unwilling or unable to return to the hospital. No doctors were included among the healthcare workers. A previous study in Bangladesh found doctors to have similar knowledge but a more positive attitude and poorer practice regarding PPE than other healthcare workers [5]. In Nigeria, doctors had better knowledge than other healthcare workers which was associated with better preventive practice [9]. Other than through participant feedback, it was not possible to clearly separate changes in KAP due to viewing the videos from those that occur for other reasons e.g. exposure to other information sources.

The study was conducted in two inpatient facilities which provide diagnostic and treatment services for COVID-19 in Bangkok. The populations attending these facilities may differ from other healthcare facilities in Thailand and from the general public outside of hospital. Being attached to a medical school and providing regular training for healthcare staff and the general public, it is likely that KAP among these groups would be better than in other locations. A much larger study would be needed to investigate this. An online questionnaire study in Bangladesh [33] found more accurate knowledge and positive attitudes with increasing age, education level, family income, and urban area residence. Among healthcare workers in a tertiary hospital in Nepal [34] appropriate practice correlated with better knowledge and a positive attitude towards COVID-19 infection was seen with increasing age.

Both the title and aims of this study included improving KAP through educational videos. From the improvements seen in this study, this appears to have been achieved. However, the first two assessments of KAP were done 3–4 months apart so it is likely that participants also learnt from other sources of information during this period. That some of the scores improved in the third assessment which was only a few days after the second suggests at least some impact from the videos. Additional evidence for people having learnt from the videos was the feedback obtained during the viewing.

The study also had strengths. The information collected from each participant was detailed and gave a nuanced understanding of areas needing improvement. The questionnaire was based on a detailed template from WHO adapted for the local context. The addition of observed practices by infection control expert nurses added another layer of evidence as well as visualising fluorescent powder on the hands of participants with standardized recording in 17 different areas. The comments and responses to the questionnaires and the observed practice on handwashing and mask wearing were used dynamically to improve the second set of videos to address the gaps in knowledge, attitude and practice.

The videos produced were quickly disseminated in Thailand by a variety of routes including the most popular social media channels. This coincided with the beginning of the second wave of transmission in the country. Being in Thai language, the videos have the major advantage of being easily accessed and understood by most of the population. With minor modifications of text, and replacement of subtitles and/or voiceovers they can also be easily adapted to other languages for use in other countries in the region and across the globe.

## Conclusion

Detailed information on gaps in knowledge, attitudes and practice among the general public and healthcare workers regarding COVID-19 transmission and its prevention in Thailand were obtained from a combination of questionnaires and observations. This was used to produce targeted educational videos which addressed these gaps with subsequent improvements on retesting. The resulting videos were then disseminated as a resource to aid in efforts to fight COVID-19 in Thailand and worldwide.

## Supplementary Information

**Additional file 1 Table S1**. Results of the statistical tests for comparisons between baseline (1) and follow up (2 and 3). *P* values shown.

## Data Availability

The data that support the findings of this study will be available upon reasonable request to MORU’s Data Access Committee [datasharing@tropmedres.ac].
